# Proportional assist ventilation relieves clinically significant dyspnea in critically ill ventilated patients

**DOI:** 10.1186/s13613-021-00958-7

**Published:** 2021-12-17

**Authors:** Côme Bureau, Maxens Decavèle, Sébastien Campion, Marie-Cécile Nierat, Julien Mayaux, Elise Morawiec, Mathieu Raux, Thomas Similowski, Alexandre Demoule

**Affiliations:** 1 Sorbonne Université, INSERM, UMRS1158 Neurophysiologie Respiratoire Expérimentale et Clinique, 75005 Paris, France; 2 AP-HP 6 Sorbonne Université, site Pitié-Salpêtrière, Service de Pneumologie, Médecine Intensive et Réanimation, Département R3S, Hôpital Pitié-Salpêtrière, 47-83 bld de l’hôpital, 75651 Paris cedex 13, France; 3 AP-HP, Groupe Hospitalier Universitaire APHP-Sorbonne Université, site Pitié-Salpêtrière, Département d’Anesthésie Réanimation, 75013 Paris, France

**Keywords:** Mechanical ventilation, Dyspnea, Clinical study, Physiology

## Abstract

**Introduction:**

Dyspnea is common and often severe symptom in mechanically ventilated patients. Proportional assist ventilation (PAV) is an assist ventilatory mode that adjusts the level of assistance to the activity of respiratory muscles. We hypothesized that PAV reduce dyspnea compared to pressure support ventilation (PSV).

**Patients and methods:**

Mechanically ventilated patients with clinically significant dyspnea were included. Dyspnea intensity was assessed by the Dyspnea—Visual Analog Scale (D-VAS) and the Intensive Care-Respiratory Distress Observation Scale (IC-RDOS) at inclusion (PSV-Baseline), after personalization of ventilator settings in order to minimize dyspnea (PSV-Personalization), and after switch to PAV. Respiratory drive was assessed by record of electromyographic activity of inspiratory muscles, the proportion of asynchrony was analyzed.

**Results:**

Thirty-four patients were included (73% males, median age of 66 [57–77] years). The D-VAS score was lower with PSV-Personalization (37 mm [20‒55]) and PAV (31 mm [14‒45]) than with PSV-Baseline (62 mm [28‒76]) (*p* < 0.05). The IC-RDOS score was lower with PAV (4.2 [2.4‒4.7]) and PSV-Personalization (4.4 [2.4‒4.9]) than with PSV-Baseline (4.8 [4.1‒6.5]) (*p* < 0.05). The electromyographic activity of parasternal intercostal muscles was lower with PAV and PSV-Personalization than with PSV-Baseline. The asynchrony index was lower with PAV (0% [0‒0.55]) than with PSV-Baseline and PSV-Personalization (0.68% [0‒2.28] and 0.60% [0.31‒1.41], respectively) (*p* < 0.05).

**Conclusion:**

In mechanically ventilated patients exhibiting clinically significant dyspnea with PSV, personalization of PSV settings and PAV results in not different decreased dyspnea and activity of muscles to a similar degree, even though PAV was able to reduce asynchrony more effectively.

**Supplementary Information:**

The online version contains supplementary material available at 10.1186/s13613-021-00958-7.

## Introduction

Dyspnea is one of the most distressing sensations experienced by critically ill patients [1]. It is estimated that one-half of intubated patients experience dyspnea, which is responsible for immediate suffering and a poorer prognosis [2]. Relief of dyspnea is a priority in these patients [3]. Dyspnea in mechanically ventilated patients is partly due to a mismatch between the patient's inspiratory effort and the level of assistance, corresponding to under-assistance [2]. This mismatch may also generate patient–ventilator asynchrony, which is associated with poorer clinical outcomes [4].

With pressure support ventilation (PSV), the most widely used mode of partial ventilatory assistance, a constant preset level of pressure assists each inspiration regardless of the patient’s inspiratory effort. A mismatch between the patient's inspiratory effort and the level of assistance is therefore likely to occur [2, 5–11]. Ventilator settings such as the assist control mode and a low pressure support level are associated with increased dyspnea [2, 12]. Proportional assist ventilation (PAV) is a mode of mechanical ventilation that adjusts the level of assistance to the intensity of respiratory muscle activity. As opposed to PSV, PAV + adjusts the level of assistance in real time to the patient's inspiratory effort, which in turn prevents under-assistance and over-assistance [13–18]. Few data are currently available concerning the impact of PAV + on dyspnea, as most studies have been conducted in healthy subjects or ICU patients without clinically significant dyspnea [13–17, 19–21].

Our first hypothesis was that PAV + would be more effective than PSV with personalized settings to relieve clinically significant dyspnea. We also hypothesized that this beneficial effect of PAV + on dyspnea would be associated with decreased respiratory drive, a major determinant of dyspnea [22] and increased variability of the breathing pattern, a marker of adequate load–capacity balance [7, 9–11, 17, 19, 20, 23]. Finally, we sought to evaluate the respective impact of PAV + and personalized PSV settings on patient–ventilator asynchrony. We therefore compared the respective impacts of PSV with personalized ventilator settings and PAV + on dyspnea in mechanically ventilated patients exhibiting clinically significant dyspnea.

## Patients and methods

This, single-center prospective study was performed over a 7-month period in the 10-bed Medical ICU of the Respiratory and ICU Division of La Pitié-Salpêtrière hospital, Paris, France.

### Patients

The study was approved by the *Comité de Protection des Personnes Ile de France VI* (No. 125-15). Informed consent was obtained from patients or relatives.

Patients were eligible when they met the following criteria: (1) intubation and mechanical ventilation for more than 24 h, for a respiratory cause with hypoxemia defined as a PaO_2_/FiO_2_ < 300 mmHg recorded at least once; (2) PSV ventilation for > 6 h; (3) clinically significant dyspnea, defined in communicative patients by a dyspnea visual analog scale (D-VAS, bounded on the left by “no respiratory discomfort” and on the right by “intolerable respiratory discomfort”) ≥ 40 on a scale from 0 to 100 or an Intensive Care-Respiratory Distress Operating Scale (IC-RDOS, see below) score ≥ 2.4 [2], and in noncommunicative patients by an IC-RDOS score ≥ 2.4; (4) decision of the physician in charge of the patient to evaluate PAV + after personalization of ventilator settings; (5) Richmond Agitation and Sedation Scale (RASS) between − 2 and + 2 and Glasgow Coma Scale > 12. Patients were considered communicative when they were able to consistently self-report dyspnea, as attested by a D-VAS variation not exceeding 10 mm for three consecutive measures [11, 24, 25].

Exclusion criteria were: (1) severe hypoxemia defined as a PaO_2_/FiO_2_ ratio < 150 mmHg; (2) delirium according to the Confusion Assessment Method for the Intensive Care Unit (CAM-ICU); (3) hemodynamic instability defined by the need for intravenous volume expansion or vasopressors during the previous 24 h; (4) age < 18 years, known pregnancy, protected adult; (5) brain damage defined by Glasgow Coma Scale < 9 and (6) neuromuscular disease.

### Measurements

Anthropometric data, medical history and current treatments were collected at inclusion.

#### Quantification of dyspnea

Dyspnea measurements were performed in the presence of two experimenters who were not the physicians in charge of the patients. Dyspnea was quantified in all patients by means of the IC-RDOS, which is an observational dyspnea scale based on five physical and observable signs of respiratory distress (heart rate, use of neck muscles during inspiration, abdominal paradox, facial expression of fear, supplemental oxygen) tailored to best correlate with D-VAS in communicative ICU patients [26]. In addition, in communicative patients only, dyspnea was also quantified by placing a cursor on a 10-cm D-VAS.

#### Airway pressure and flow

Airway flow was measured with a pneumotachograph (Hans Rudolph, Kansas City, USA) inserted between the Y-piece and the endotracheal tube before being connected to a differential pressure transducer (Validyne, Northridge, USA). Airway pressure was measured at the Y-piece by a differential pressure transducer (Validyne, Northridge, USA).

#### Electromyography (EMG) of extradiaphragmatic inspiratory muscles

The amplitude of the extradiaphragmatic inspiratory muscle EMG signal is a surrogate for central respiratory drive and is proportional to dyspnea intensity [27]. The EMG signal was collected by self-adhesive surface electrodes like those commonly used to record the electrocardiogram signal in critically ill patients, with an interval of 2 cm between the two electrodes. Electrode positions varied according to the muscle recorded. For EMG of the parasternal intercostal muscles, electrodes were placed next to the second intercostal space as close as possible to the sternum. For EMG of the alae nasi muscles, electrodes were placed on the lateral surfaces of the nose (nostrils) [12]. EMG signals were amplified, sampled at a frequency of 10 kHz, filtered between 40 and 500 Hz (PowerLab, AD Instruments, Hastings, UK) and the root-mean-squared electromyogram was calculated [12, 28] (see Additional file 1: method, detailed measurement of electromyography of extradiaphragmatic inspiratory muscles).

#### Arterial blood gases

Blood gases were sampled at the end of each condition using an arterial catheter.

### Study design

Patients were mechanically ventilated by a PB 840 ventilator (Medtronic, Boulder, USA). Three successive conditions were studied. Changes in settings were decided by the physician in charge of the patient.

The first condition was defined as "PSV-Baseline" and corresponded to the settings used at the time of inclusion by the physician in charge. These ventilator settings were those observed on the ventilator when significant dyspnea was detected. The second condition was defined as "PSV-Personalization" and consisted in personalization of PSV settings left to the discretion of the physician in charge of the patient in order to reduce clinically significant dyspnea. Ventilator adjustments were applied to at least one setting among pressure support level, cycling-off and inspiratory trigger. Briefly, the level of pressure support was increased and the level of expiratory trigger was decreased in order to increase the level of assistance without generating a tidal volume greater than 10 mL/kg or inducing ineffective triggering, corresponding to asynchrony that is known to be associated with over-assistance [29, 30]. The third condition was defined as “PAV”. The patient was first switched to the PAV + mode. As recommended by local guidelines with this mode, the level of assistance, referred to as assistance percentage or gain, was set to maintain the patient at a respiratory effort target corresponding to a muscle pressure–time product (PTPmus) between 50 and 150 cmH_2_O·s·min^−1^. As it was not possible to directly calculate PTPmus at the bedside, we analyzed the maximum inspiratory muscle pressure, considering it to be the main component of PTPmus, as previously reported [31]. The peak respiratory muscle pressure was estimated as (peak airway pressure − positive end-expiratory pressure (PEEP) × ((100 − gain)/gain). A grid constructed from this equation provided a quick estimation of the peak respiratory muscle pressure at the bedside, which was maintained between 5 and 10 cmH_2_O [31] (see Additional file 1: Method, Algorithm for PAV + adjustment).

Each patient underwent three 30-min trials under each condition, consisting of a 20-min initial stabilization period followed by a 10-min recording. The endotracheal tube was suctioned before starting each recording. PEEP and FiO_2_ were maintained constant throughout the study period at the values used before patient enrolment.

### Data analysis

#### Breathing pattern and breath-by-breath variability

Breathing pattern variables were determined on a breath-by-breath basis for each of the three conditions (Labchart 7.3® software, ADInstruments, Dunedin, New Zealand). These included respiratory rate (RR), tidal volume (Vt), inspiratory time (Ti), and maximal inspiratory pressure (Pmax). The coefficient of variation for RR, Vt, Ti, and Pmax were calculated as the ratio of the standard derivation to the mean.

#### Detection and quantification of patient–ventilator asynchrony

The three most common observed forms of patient–ventilator asynchrony were quantified on the basis of flow and pressure on the 10-min recording preformed in each condition [32]. Ineffective triggering (IT) was defined as an airway pressure drop > 0.5 cmH_2_O or flow elevation not followed by a ventilatory cycle. Double triggering (DT) was defined as the presence of two ventilatory cycles separated by a very short expiratory time. Auto-triggering (AT) was defined as a ventilatory cycle without a prior pressure drop [13]. The asynchrony index (AI) was defined as ([AT + IT + DT]/total respiratory rate) × 100; total respiratory rate was defined as [IT + respiratory rate] [13] (see Additional file 1: Method S3. Quantification of patient–ventilator asynchrony).

#### Electromyogram (EMG) of extradiaphragmatic inspiratory muscles

Inspiratory muscle EMG activity, used to estimate respiratory drive, was processed using the Labchart Peak Analysis module MLS380/8 (ADInstruments, Dunedin, New Zealand). This module generated a root mean square of the EMG smoothed over 1-s fixed windows that was used to measure the maximum amplitude of the EMG signal (EMGmax), defined by the difference between the maximum amplitude of the root mean square signal (RMS) and its baseline, and the area under the EMG signal curve (EMGauc), calculated by integration of the RMS between its deflection from the baseline and its return to this same baseline time-locked on a respiratory cycle [28]. EMGmax and EMGauc were expressed as a proportion of the activity measured under the "PSV-Baseline" condition in order to normalize the values due to the variability of skin impedance and patient's morphology.

### Statistical analysis

Statistical analysis was performed with Prism 8.0 software (GraphPad Software, USA). Kolmogorov–Smirnov test was used to assess normality of data distribution. Quantitative variables were described by their median and interquartile interval. Qualitative variables were expressed in absolute value and percentage. All analyses were performed with a type I error of 5%. Results were compared between the following conditions: PSV-Baseline, PSV-Personalization and PAV. Discrete variables were compared by Chi-square test. Continuous variables were compared using a Friedman test, followed, when positive, by a post hoc comparison using a Dunn multiple comparison test. Correlations between variables were evaluated using the Spearman rank correlation coefficient.

Considering a power of 80% and standard deviations of 3 for IC-RDOS and 10% for the patient–ventilator asynchrony index, we calculated a sample size of at least 28 patients for this study. As we expected that PSV-Personalization or PAV could not be achieved in 20% of patients, we increased sample size to 34 patients.

## Results

Ninety-five patients with invasive mechanical ventilation and dyspnea were admitted during the period. One patient was under 18 years, six with RASS + 2, 19 refused to participate, for 35 a technical reason (equipment failure, weekend) could not allow inclusion.

Thirty-four patients were included. Patient characteristics are shown in Table 1. Thirty-three patients were connected to the ventilator using an endotracheal tube, while one patient was connected using a tracheostomy tube. The three conditions were studied in all patients. Eight patients were still receiving sedation at the time of inclusion, they were all receiving sufentanil at a dose of 5 µg/h. Ventilator settings in each of the three conditions are reported in Table 2. Personalization consisted in decreasing the inspiratory trigger for 13 patients (38%), increasing the pressure support level for all patients and decreasing the cycling-off for 18 patients (53%). Compared to PSV-Baseline, pressure support level was higher and cycling-off level was lower after personalization of ventilator settings. FiO_2_ and PEEP were not significantly different across the three conditions.

**Table 1:** Characteristics of the study population

*Demographic characteristics*	
Gender, male	25 (73)
Age, year	66 (57‒77)
Weight, kg	75 (64‒91)
Height, m	1.72 (1.61‒1.78)
Body mass index, kg m^−2^	26 (22‒29)
Duration of mechanical ventilation prior to inclusion, days	6 (4‒9)
Duration of mechanical ventilation after inclusion, days	3 (2‒3)
At least one spontaneous breathing trial performed before enrolment, *n* (%)	7 (21)
Sedation on inclusion, *n *(%)	8 (23)
RASS	0.0 (-0.7‒1.0)
*Comorbidities*	
Chronic respiratory disease, *n *(%)	14 (41)
Chronic heart failure, *n *(%)	2 (6)
*Reason for intubation*	
Bacterial pneumonia, *n* (%)	11 (33)
Viral or fungal pneumonia, *n* (%)	4 (12)
Aspiration pneumonia, *n *(%)	3 (9)
Acute on chronic respiratory failure, *n *(%)	10 (30)
Cardiogenic pulmonary edema, *n* (%)	2 (6)
Other, *n* (%)	4 (12)
*Severity scores*	
SAPS at admission	56 (38‒66)
SOFA at inclusion	6 (4‒9)

*RASS* Richmond Agitation and Sedation Scale, *SAPS* Simplified Acute Physiology Score, *SOFA* Sepsis-related Organ Failure Assessment

Data are expressed as median (interquartile range) and number (%)

**Table 2:** Ventilator settings at baseline (PSV-Baseline), after personalization of ventilator settings (PSV-Personalization) and with proportional assist ventilation (PAV)

	PSV-Baseline	PSV-Personalization	PAV
FiO_2_*, %*	30 (30‒40)	30 (30‒40)	30 (30‒40)
PEEP*, cmH*_*2*_*O*	5 (5‒6)	5 (5‒6)	5 (5‒6)
Pressure support level*, cmH*_*2*_*O*	6 (6‒8)	14 (14‒16)*	‒
Inspiratory trigger*, L/min*	1 (1‒2)	1 (1‒1)	‒
Cycling off, *%*	30 (25‒30)	20 (15‒29)*	‒
Percentage assist*, %*	‒	‒	70 (70‒80)
Flow sensitivity*, L/min*	‒	‒	2.0 (1.5‒2.0)
Expiratory sensitivity*, L/min*	‒	‒	2 (1‒2)

*FiO*_*2*_ inspired oxygen fraction, *PEEP* positive end-expiratory pressure

Data are expressed as median (interquartile range) and number (%)

*p < 0.05 as compared to PSV-Baseline

### Dyspnea and extradiaphragmatic inspiratory muscle EMG activity

**Fig. 1:**
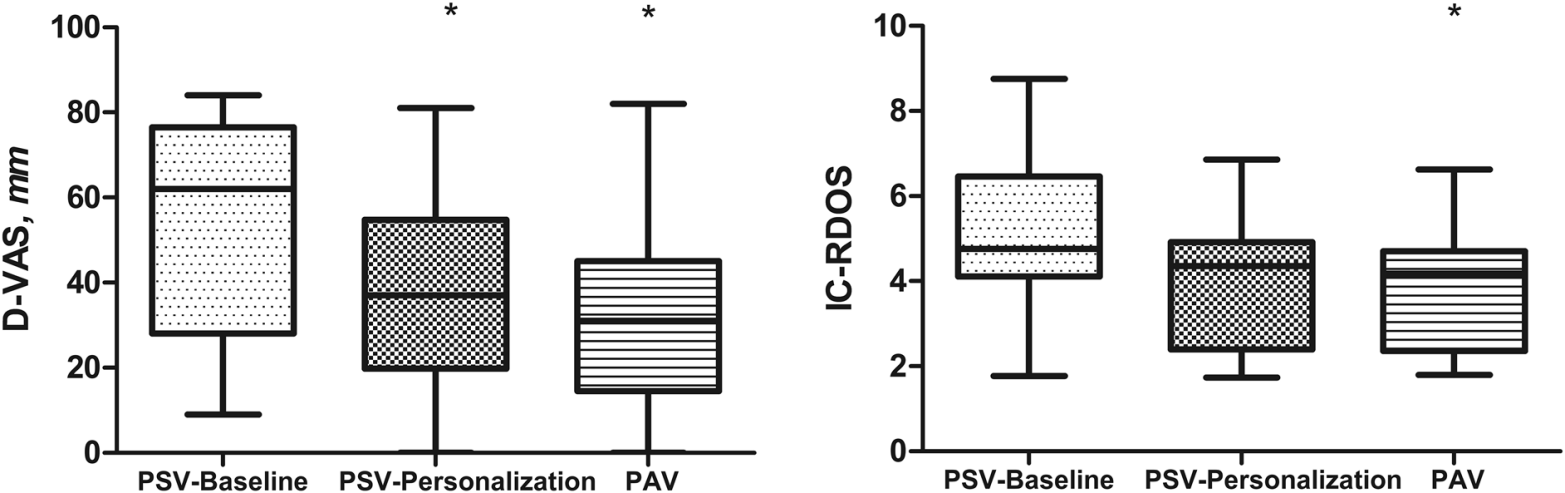
Dyspnea visual analog scale (D-VAS) and Intensive Care-Respiratory Distress Observation Scale (IC-RDOS) at baseline (PSV-Baseline), after personalization of ventilator settings (PSV-Personalization) and with proportional assist ventilation (PAV). *Line inside the boxes* median, *limits of the boxes* 75th and 25th percentiles of the data (interquartile range), *whiskers* 5th and 95th percentiles. * p < 0.05 compared to PSV-Baseline

D-VAS scores for communicative patients and IC-RDOS scores for all patients in the three conditions are shown in Fig. 1. D-VAS could not be rated in 16 patients due to hearing impairment (*n* = 3), language barriers (*n* = 4); ongoing sedation (*n* = 4) and misunderstanding of the instructions (*n* = 5). D-VAS was therefore available in 18 patients. D-VAS was lower with PSV-Personalization (37 [20‒55], *p* = 0.001) and PAV (31 [14–45], *p* = 0.001) than with PSV-Baseline (62 [28‒76], *p* = 0.001 for both). There was no significant difference in terms of D-VAS between PSV-Personalization and PAV. IC-RDOS was measured in all patients. IC-RDOS was lower with PAV (4.18 [2.36‒4.71]) than with PSV-Baseline. (4.76 [4.11‒6.46], *p* = 0.002), but there was no significant difference with PSV-Personalization (4.35 [2.39‒4.92]. There was no significant difference between PSV-Personalization and PAV. The results are expressed as differences between the conditions in Additional file 1: Table S1.

In communicative patients (*n* = 18) there was a significant positive correlation between D-VAS and IC-RDOS with PSV-Personalization (Rho = 0.54 [0.07–0.81], *p* = 0.02), but there was no significant correlation with PSV-Baseline (Rho = 0.41 [− 0.10 to 0.75], *p* = 0.10) and PAV (Rho = 0.44 [− 0.06 to 0.77], *p* = 0.07) (see Additional file 1: Figure S1).

There was a significant negative correlation between D-VAS and the coefficient of variation of RR (Rho = − 0.29 [− 0.52 to − 0.02], *p* = 0.031) (see Additional file 1: Figure S2). There was a significant negative correlation between IC-RDOS and the coefficient of variation of RR (Rho =− 0.43 [− 0.58 to − 0.26], *p* < 0.0001), Vt (Rho = − 0.33 [− 0.50 to − 0.15], *p* = 0.001), Ti (Rho = − 0.22 [− 0.40 to − 0.02], *p* = 0.025) and Pmax (Rho =− 0.33 [− 0.50 to − 0.14], *p* = 0.001).

There was a significant negative correlation between IC-RDOS and the pH in PSV (Rho = − 0.51 [− 0.71 to − 0.24], *p* = 0.001), but there was no correlation in PAV (Rho = − 0.29 [− 0.66 to − 0.19], *p* = 0.221). There was no correlation between IC-RDOS and bicarbonatemia in PSV (Rho = − 0.14 [− 0.43 to 0.18], *p* = 0.371) or in PAV (Rho = − 0.08 [− 0.52 to 0.39], *p* = 0.734) Additional file 1: Figure S3).

**Fig. 2:**
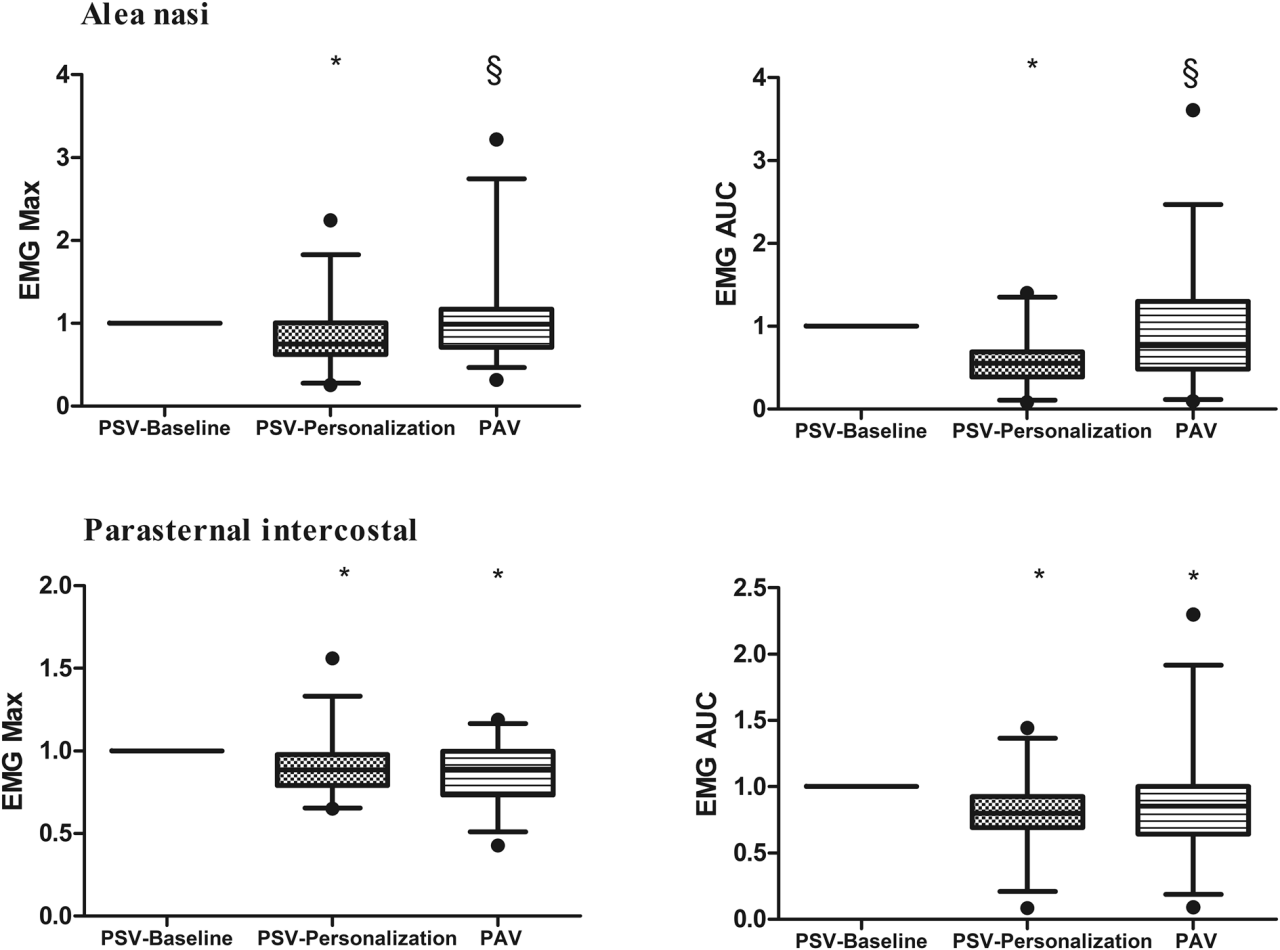
Electromyographic (EMG) activity of *Alae nasi* and parasternal intercostal muscles at baseline (PSV-Baseline), after personalization of ventilator settings (PSV-Personalization) and with proportional assist ventilation (PAV). The EMG activity is described by peak EMG (EMGmax) and area under the curve (EMGauc) and is expressed as a proportion of the EMG activity under the PSV-Baseline condition. *Line inside the boxes* median, *limits of the boxes* 75th and 25th percentiles of the data (interquartile range), *whiskers* 5th and 95th percentiles. * p < 0.05 compared to the PSV-Baseline condition, § p < 0.05 compared to the PSV-Personalization condition

EMG activity of the *Alae nasi* and parasternal intercostal muscles in the three conditions is shown in Fig. 2. Compared to PSV-Baseline, *Alae nasi* EMG activity was lower with PSV-Personalization (*p* = 0.008). *Alae nasi* EMG activity was higher with PAV than with PSV-Personalization, but there was no significant difference in terms of *Alae nasi* EMG activity between PSV-Baseline and PAV. Compared to PSV-Baseline, parasternal intercostal muscle EMG activity was lower with PSV-Personalization and PAV (*p* = 0.001). There was no significant difference in terms of parasternal intercostal EMG activity between PSV-Personalization and PAV.

### Breathing pattern, breath-by-breath variability, and blood gases

Breathing pattern, coefficients of variation and blood gases are shown in Table 3. Compared to PSV-Baseline, PSV-Personalization of ventilator settings resulted in a decrease of RR and an increase of Ti, Vt and Vt/ Ti. Compared to PSV-Personalization, PAV resulted in an even lower RR, higher Ti, and lower Vt/Ti.

**Table 3:** Breathing pattern and blood gases at baseline (PSV-Baseline), after personalization of ventilator settings (PSV-Personalization) and with proportional assist ventilation (PAV)

	PSV-Baseline	PSV-Personalization	PAV	***p***
*Breathing pattern*				
RR, min^‒1^	27 (22‒32)	24 (20‒30)*	21 (17‒28)*§	< 0.0001
*V*t, ml/kg IBW	7.0 (5.6‒9.7)	7.8 (7.0‒9.8)*	9.0 (6.5‒10.5)*	< 0.0001
*T*i, sec	0.75 (0.61‒0.95)	0.85 (0.67‒1.03)	0.97 (0.82‒1.27)*§	< 0.0001
*V*t/ *T*i, L/s^‒1^	1.15 (0.74‒1.55)	1.97 (1.23‒3.16)*	0.84 (0.62‒1.06)§	< 0.0001
*P*max, cmH_2_O	13 (12‒15)	21 (18‒23) *	24 (19‒29)*	< 0.0001
EtCO_2,_ mmHg	33 (28‒38)	31 (26‒35)	31 (27‒37)	0.033
*Breath-by-breath variability*				
CV RR, %	13 (10–20)	14 (11–24)	22 (15–28) * §	0.001
CV Vt, %	15 (11–20)	15 (10–26)	25 (17–43) * §	< 0.001
CV Ti, %	22 (16–43)	33 (19–50)	22 (13–27) §	0.023
CV Vt/ Ti, %	23 (16–39)	29 (15–42)	23 (14–60)	0.632
CV Pmax, %	4 (4–06)	6 (3–10)	16 (11–23) * §	< 0.001
*Blood gases*				
pH	7.43 (7.36‒7.44)	7.41 (7.39‒7.47)*	7.42 (7.37‒7.48)*	0.012
PaO_2,_ mmHg	80 (73‒99)	87 (73‒101)	88 (79‒98)*	0.016
PaCO_2,_ mmHg	41 (36‒49)	39 (34‒47)*	39 (33‒48)	0.001
SaO_2_, %	96 (94‒98)	96 (94‒98)	97 (96‒98)	0.038
HCO^3−^_,_ mmol/L	26.6 (22.7‒28.9)	26.8 (22.7‒29.4)	26.9 (22.1‒28.6)	0.503
A-aO_2_ gradient, mmHg	94 (72‒154)	96 (85‒158)	82 (64‒143) §	0.023

*RR* respiratory rate, *Ti *inspiratory time, *Vt* tidal volume, *IBW* ideal body weight, *EtCO*_*2*_ CO_2_ expired fraction, *CV* coefficient of variation, *Pmax* peak airway pressure, *A-aO*_*2*_ Gradient, alveolar–arterial oxygen gradient

Data are expressed as median (interquartile range) and number (%)

* *p* < 0.05 compared to the PSV-Baseline condition; § *p* < 0.05 compared to the PSV-Personalization condition

The coefficients of variation of RR, Vt, and Pmax were higher with PAV than with PSV-Baseline and PSV-Personalization.

pH was lower with PSV-Personalization and PAV than with PSV-Baseline. PaO_2_ was higher with PAV than with PSV-Baseline and PaCO_2_ was lower with PSV-Personalization than with PSV-Baseline.

The results are expressed as differences between the conditions in Table S1.

### Patient–ventilator asynchrony

**Fig. 3:**
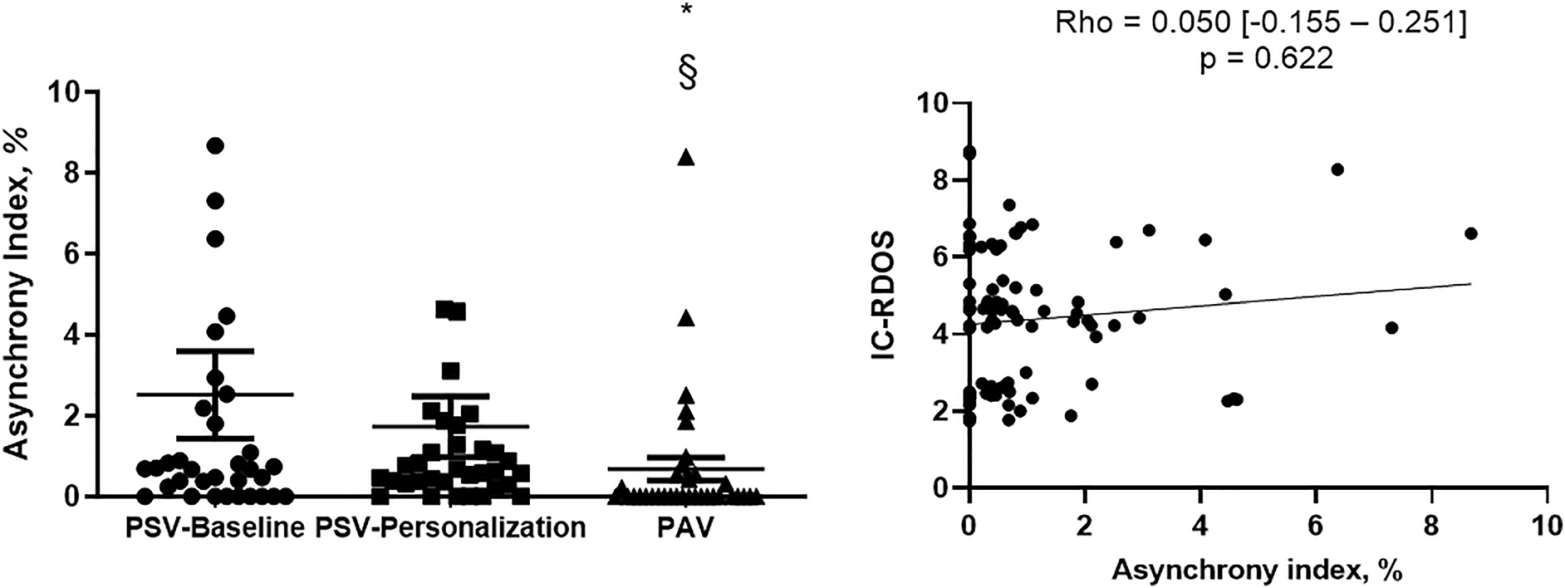
Individual asynchrony index (A) at baseline (PSV-Baseline), after personalization of ventilator settings (PSV-Personalization) and with proportional assist ventilation (PAV); correlation between the individual asynchrony index and Intensive Care-Respiratory Distress Observation Scale (IC-RDOS) (B). The horizontal solid line indicates median value. * p < 0.05 compared to PSV-Baseline, § p < 0.05 compared to PSV-Personalization

The AI under the three conditions is shown in Fig. 3. There was no significant difference between the AI with PSV-Baseline (0.68 [0‒2.28]%) and PSV-Personalization (0.60 [0.31‒1.41]%,). The AI was lower with PAV (0 [0‒0.55]%) than with PSV-Baseline (*p* = 0.001) and PSV-Personalization (*p* = 0.001). There was no correlation between the AI and dyspnea assessed by IC-RDOS (Rho = 0.05 [− 0.16 to 0.25], *p* = 0.622) (Fig. 3). The decreased prevalence of double triggering accounted for most of the reduction of AI observed with PAV, as PAV was not associated with a decrease of the prevalence of auto-triggering or ineffective triggering (see Additional file 1: Figure S4). There was no correlation between the double triggering prevalence and dyspnea assessed by IC-RDOS (Rho = 0.01 [− 0.20 to 0.21], *p* = 0.953) (see Additional file 1: Figure S5).

## Discussion

Our results can be summarized as follows. In patients with clinically significant dyspnea: (1) PAV + and personalized PSV settings relieved dyspnea; (2) PAV + and personalized PSV settings seemed not to have a different impact on dyspnea and respiratory drive, as assessed by respiratory muscle EMG activity; (3) breath-by-breath variability was higher with PAV + and was inversely correlated with dyspnea; (4) PAV + was more effective than personalized PSV settings to reduce patient–ventilator asynchrony. To our knowledge, this is the first study to compare personalized PSV settings and PAV + in mechanically ventilated patients exhibiting clinically significant dyspnea. In this study, we confirm that simple changes in ventilator settings can suffice to achieve a spectacular effect on dyspnea [2]. In addition, we have increased the panel of possible changes of ventilator settings by adding another option, which is switching to PAV + .

### Dyspnea, variability, load–capacity balance and neural drive to breathe

Our results suggest that improvement of dyspnea resulted from a reduction of respiratory loading, which in turn reduced the intensity of respiratory drive, as illustrated by the decreased amplitude of the parasternal intercostal and *alae nasi* EMG activities, two surrogates for central respiratory drive. These findings are in complete agreement with previous studies reporting a close correlation between dyspnea and respiratory drive in mechanically ventilated patients, and a cortical contribution to the drive to breathe in dyspneic MV patients [12, 33]. Improvement of dyspnea was associated with a concomitant increase in *V*t, which raises an issue regarding protective mechanical ventilation with low *V*t in patients able to perceive dyspnea. Indeed, low *V*t is recommended for adequate lung protection, but seems to be associated with more severe respiratory suffering [7]. The increase in breath-by-breath variability is another argument in favor of reduction of respiratory loading with PAV + . In awake normal humans, breath-to-breath variability tends to decrease in response to mechanical loading [34]. During weaning from mechanical ventilation, an inverse relationship between the breath-to-breath variability of *V*t/*T*i and dynamic compliance of the respiratory system has been reported [35]. In contrast, unloading of the respiratory system with increasing levels of assistance increases breath-by-breath variability [36]. The load–capacity relationship of the respiratory system is therefore a major determinant of breath-to-breath variability: the higher the loading, the lower the variability. However, the higher breath-by-breath variability observed with PAV + may also be due to the proportionality of the assistance that “unmasks” the underlying variability in central respiratory neural output [36]. In other words, PAV + reveals the natural fluctuations in the resting breathing pattern of humans that appear to originate from the activity of central pattern generators [37]. This proportional ventilation, which tends to preserve the natural variability of breathing, may also explain why PAV + is slightly more beneficial on dyspnea. To the best of our knowledge, this is the first report of an inverse correlation between dyspnea and breath-by-breath variability.

### Patient–ventilator asynchrony

While personalized PSV failed to decrease patient–ventilator asynchrony, PAV + significantly decreased the prevalence of asynchrony and, more specifically, the prevalence of double triggering [38], a pattern of asynchrony that may increase tidal volume and consequently expose the lung to overdistension and subsequent ventilator-induced lung injury [39, 40]. In addition, double triggering is a form of asynchrony that occurs when the level of assistance is too low and does not meet a high respiratory drive [32]. The reduction of double triggering provides further evidence that PAV + is associated with a better load–capacity balance.

### Clinical implications

Although PAV + and personalized PSV were equally effective on dyspnea, it should be stressed that they are not equally time-consuming. On the one hand, personalization of PSV is very time-consuming, as it requires regular assessment of the breathing pattern to better adapt ventilator settings. On the other hand, PAV + constantly adapts the level of assistance delivered by the ventilator to the patient's inspiratory muscle activity, without any human intervention. Indeed, in a study that evaluated to what extent mechanical ventilation with PAV + could keep patients in a comfort zone for several days, the median daily number of load-adjustable gain factors was one [31]. The fact that PAV + was more effective than PSV to reduce the prevalence of patient–ventilator asynchrony is another argument supporting a potential benefit of PAV + , as asynchrony is associated with prolonged duration of mechanical ventilation and even high mortality [4, 41].

### Strengths and limitations of the study

A major strength of our study is that it compared PAV + delivered according to a standard protocol to PSV with improved settings. In previous studies comparing PAV + and PSV, PSV was delivered in the absence of any precise guidelines and it was therefore difficult to conclude whether the benefit of PAV + was actually due to superiority of this mode of ventilation per se or to poorly personalized PSV settings. In addition, PAV + was also delivered according to a protocol designed to maintain the patient within a so-called “comfort zone” that corresponds to a oesophageal pressure time between 50 and 150 cmH_2_O·s·min^−1^ [31]. Another strength of the study is that it focused on dyspnea, which is clearly a major cause of suffering for ICU patients [7]. It must be stressed that relief of dyspnea is an essential clinical objective, which, like pain, is currently considered by some authors to be a basic human right [42, 43]. Here, the change from PSV-Baseline to either PSV-Personalization or PAV was associated with a median change > 10 mm, which is considered as the minimal clinically important difference (MCID) for D-VAS [44, 45]. In addition, dyspnea may be associated with difficult weaning and increased duration of mechanical ventilation [2]. Finally, dyspnea may be associated with unpleasant recollections following the ICU stay [9] and may contribute to the pathogenesis of post-traumatic stress disorder [11].

This study also presents a number of limitations. First, PSV was personalized according to the clinical judgment of the physician in charge of the patient. This personalization led to a conflict between the need to protect the diaphragm from over-assistance and the desire to relieve the brain from dyspnea. It is observed that even with a normal Pmus and a comfort zone in PAV + , there is a risk of over-assistance (tidal volume 9 ml/kg IBW, 70% of percentage assist). The non-randomized order of the interventions may have affected the results but the aim was to reproduce the same sequence of care as in real life; first, to personalize settings of reference mode and only this personalization fails, then change the ventilatory mode. This study evaluated only the short-term effects of changing ventilatory mode on dyspnea sensation, not the long-term effects. Subsequently, we cannot determine whether the effect of higher levels of support on dyspnea is maintained beyond this interval. Personalization based on esophageal pressure time or work of breathing may have been more accurate, but it would have required invasive measurements that cannot be transposed to daily practice. For the same reason, we did not measure the EMG activity of the diaphragm, which also requires invasive techniques. On the contrary, we focused on the EMG activity of parasternal intercostal and *Alae nasi muscles*, which can be measured noninvasively and which has been shown to be a reliable marker of respiratory drive and subsequent dyspnea [12]. Second, detection and management of patient–ventilator asynchrony was based on visual inspection of the flow and pressure trace because we wanted the study to be as less invasive as possible. Although, previous studies have suggested that, when analyzing flow and pressure signals, physicians may not be able to detect up to two-thirds of episodes of patient–ventilator asynchrony [46, 47], other studies have shown this technique to be reliable [13]. Third, it is not possible to know whether the slight discrepancy between the changes in parasternal intercostal and *Alae nasi* EMG activities is related to a technical issue with the recording of the parasternal intercostal activity (see Additional file 1: Method S1**)** or to a lower central respiratory drive to the parasternal intercostal muscles. Finally, we decided to include also patients with poor communication in order to obtain a population that reflects the reality of daily practice in the ICU. Similarly, we included patients with a low dose of sedation at the time of dyspnea assessment to approximate real life. We were only able to perform a self-assessment of dyspnea in half of the subjects, which may have influenced the results.

## Conclusion

In patients with clinically significant dyspnea, the PAV + mode improves dyspnea as well as patient–ventilator asynchrony. The parallel decrease in inspiratory muscle EMG activity and the increase in breath-to-breath variability are consistent with and provide a physiological dimension to these findings. In this study, this benefit of PAV + on dyspnea was fairly comparable to the benefit of personalization of ventilator settings in PSV. However, it should be stressed that personalization of ventilator settings in PSV must be regularly re-evaluated, which is a time-consuming procedure. This is not the case with PAV + , which constantly adapts the level of assistance delivered by the ventilator to the patient's needs. The present study paves the way to future trials investigating the benefit of PAV + on dyspnea in larger populations.

## Supplementary Information

**Additional file 1. Method S1.** Detailled measurement of electromyography of extradiaphragmatic inspiratory muscles. **Method S2.** Quantification of patient-ventilator asynchrony. **Method S3. **Algorythm for PAV + adjustement. **Table S1.** Differences of dyspnea, breathing pattern and blood gases between baseline (PSV-Baseline), after optimization of ventilator settings (PSV-Personalization) and with proportional assist ventilation (PAV). **Figure S1.** Correlation between the dyspnea visual analog scale (D-VAS) and Intensive Care Respiratory Distress Observation Scale (IC-RDOS) in communicative patients (n = 18) at baseline (PSV-Baseline), after optimization of ventilator settings (PSV-Optimization) and with proportional assist ventilation (PAV). **Figure S2.** Correlation between the coefficient of variation (CV) of descriptors of the breathing pattern and dyspnea assessed by the dyspnea visual analog scale (D-VAS, left panels) and Intensive Care Respiratory Distress Observation Scale (IC-RDOS, right panels). **Figure S3.** Correlation between the pH and Intensive Care Respiratory Distress Observation Scale (IC-RDOS) in pressure support ventilation (left) and proportional assisted ventilation (right panel). **Figure S4.** Prevalence of double triggering (Panel A), prevalence of auto-triggering (Panel B) and prevalence of ineffective triggering (Panel C) at baseline (PSV-Baseline), after personalization of ventilator settings (PSV-Personalization) and with proportional assist ventilation (PAV). **Figure S5.** Correlation between the prevalence of double triggering and Intensive Care Respiratory Distress Observation Scale (IC-RDOS).

## Data Availability

The datasets analyzed during the current study are available from the corresponding author on reasonable request.
