# Supplementary material for: Proportional assist ventilation relieves clinically significant dyspnea in critically ill ventilated patients
Source: Ann Intensive Care. 2021 Dec 17;11:177. doi: 10.1186/s13613-021-00958-7 (PMC8683518; doi:10.1186/s13613-021-00958-7)
Supplement: Supplementary file 1 — Additional file 1. Method S1. Detailled measurement of electromyography of extradiaphragmatic inspiratory muscles. Method S2. Quantification of patient-ventilator asynchrony. Method S3. Algorythm for PAV + adjustement. Table S1. Differences of dyspnea, breathing pattern and blood gases between baseline (PSV-Baseline), after optimization of ventilator settings (PSV-Personalization) and with proportional assist ventilation (PAV). Figure S1. Correlation between the dyspnea visual analog scale (D-VAS) and Intensive Care Respiratory Distress Observation Scale (IC-RDOS) in communicative patients (n = 18) at baseline (PSV-Baseline), after optimization of ventilator settings (PSV-Optimization) and with proportional assist ventilation (PAV). Figure S2. Correlation between the coefficient of variation (CV) of descriptors of the breathing pattern and dyspnea assessed by the dyspnea visual analog scale (D-VAS, left panels) and Intensive Care Respiratory Distress Observation Scale (IC-RDOS, right panels). Figure S3. Correlation between the pH and Intensive Care Respiratory Distress Observation Scale (IC-RDOS) in pressure support ventilation (left) and proportional assisted ventilation (right panel). Figure S4. Prevalence of double triggering (Panel A), prevalence of auto-triggering (Panel B) and prevalence of ineffective triggering (Panel C) at baseline (PSV-Baseline), after personalization of ventilator settings (PSV-Personalization) and with proportional assist ventilation (PAV). Figure S5. Correlation between the prevalence of double triggering and Intensive Care Respiratory Distress Observation Scale (IC-RDOS). [file 13613_2021_958_MOESM1_ESM.docx]

**Proportional assist ventilation relieves clinically significant dyspnea in critically ill ventilated patients**

Côme Bureau, MD, Maxens Decavèle, MD, Sébastien Campion, MD, PhD, Marie-Cécile Nierat, PhD, Julien Mayaux, MD, Elise Morawiec, MD, Mathieu Raux, MD, PhD, Thomas Similowski, MD, PhD, Alexandre Demoule, MD, PhD

**Additional file**

**Method**

- **Detailled measurement of electromyography of extradiaphragmatic inspiratory muscles**
- **Method 2. Quantification of patient-ventilator asynchrony**
- **Method 3. Algorythm for PAV + adjustement**

**Additional table**

- **Table S1. Differences of dyspnea, breathing pattern and blood gases between baseline (PSV-Baseline), after optimization of ventilator settings (PSV-Personalization) and with proportional assist ventilation (PAV)**

**Additional figures**

- **Figure S1. Correlation between the dyspnea visual analog scale (D-VAS) and Intensive Care Respiratory Distress Observation Scale (IC-RDOS) in communicative patients (n = 18) at baseline (PSV-Baseline), after optimization of ventilator settings (PSV-Optimization) and with proportional assist ventilation (PAV)**
- **Figure S2. Correlation between the coefficient of variation (CV) of descriptors of the breathing pattern and dyspnea assessed by the dyspnea visual analog scale (D-VAS, left panels) and Intensive Care Respiratory Distress Observation Scale (IC-RDOS, right panels)**
- **Figure S3. Correlation between the pH and Intensive Care Respiratory Distress Observation Scale (IC-RDOS) in pressure support ventilation (left) and proportional assisted ventilation (right panel)**
- **Figure S4. Prevalence of double triggering (Panel A), prevalence of auto-triggering (Panel B) and prevalence of ineffective triggering (Panel C) at baseline (PSV-Baseline), after personalization of ventilator settings (PSV-Personalization) and with proportional assist ventilation (PAV)**
- **Figure S5. Correlation between the prevalence of double triggering and Intensive Care Respiratory Distress Observation Scale (IC-RDOS)**

**Additional methods**

***Detailled measurement of electromyography of extradiaphragmatic inspiratory muscles***

Collecting phasic EMG activity from the neck muscles is not straightforward. Collection by needle electrodes provides good quality signals, but the invasive nature of this collection technique precludes its use in a clinical context. The surface electrodes are in no way invasive, but the collection of the phasic activity of the neck muscles is complex given the low amplitude of the EMG signal. As a result, the signal-to-noise ratio of the phasic EMG activity of the neck muscles collected by surface electrodes is low. On the other hand, it is well shown that the averaging of the EMG signal on the surface of the scalenes makes it possible to optimize the signal-to-noise ratio (1). This method could be applied to other extradiaphragmatic inspiratory muscles such as the parasternal intercostal muscles (2) or the wings of the nose (3).

Our team has developed an alternative acquisition technique for this activity, using surface electromyography, which is non-invasive and thus achievable in clinical practice (4). Although this is an average of the electromyographic surface activity, the latter is proportional to the level of assistance that the patients need, thus testifying to the relevance of this approximation.

The primary cause of differences in impedance is the different property of the skin between individuals (5). Studies have shown that thermal noise can account for as little as 10% of the electrode-skin noise signal (5). The noise from the electrolyte-skin interface can then be estimated from measures of noise taken from recordings on the skin, and has been shown to vary between 1 and 15 μV (6).

***Additional method 2. Algorythm for PAV + adjustement***

PAV+ is based on the equation of motion of the respiratory system. This equation relates the flow and volume entering the system at each point in the cycle to the total pressure required, linked to the respiratory mechanics : ((Ptot = P0 + R. V’ + E. V.), Ptot : total pressure (cmH2O) ; P0 : start pressure (cmH2O) ; R : resistance (cmH2O/l. sec) ; E : elastance (cmH2O/L) ; V’ : airway flow (l/s) ; V : volume (L)). Total pressure is the sum of the airway pressure (Paw), which is the assist pressure delivered by the ventilator, and the muscle pressure (Pmus) generated by the patient's breathing : Ptot = Paw + Pmus. The ventilator automatically measures the elastance and resistance of the respiratory system by means of 300 millisecond teleinspiratory microocclusions. These measurements are randomised every four to ten cycles. With the elastance and resistance of the system known, the ventilator, by measuring the instantaneous flows and volumes, can calculate the value of the total pressure at each instant. The assistance delivered is then a percentage of the total pressure, and this percentage corresponds to the gain : Paw = Gain. (R . V’ + E . V) = Gain . Ptot. Thus, in PAV+, support is proportional to the instantaneous flow and volume and the load on the system, and therefore to the total pressure. The proportionality factor is the set gain, which is the percentage of the total pressure handled by the ventilator, the rest being handled by the patient's breathing muscles.

The airway pressure delivered by the ventilator in PAV+ is therefore directly proportional to the instantaneous muscle pressure of the patient. The only proportionality factor in the equation is the set gain : Paw = (Gain/[1 – Gain]) . Pmus.

As PAV+ offers the possibility of monitoring a reproducible respiratory effort index, we used the simple gain adjustment modality to conduct weaning in PAV+ (7).

The gain adjustment protocol during PAV+ ventilation was that of Carteaux et al. and was designed to keep the patient within a reasonable target range of respiratory effort, which we defined as a respiratory muscle pressure-time product (PTPmus) between 50 and 150 cm H2O-s/min. As PTPmus cannot be calculated at the bedside, we used its main component as a surrogate: the peak muscle pressure of the respiratory muscles (Pmus,Peak). This pressure is the maximum swing of the inspiratory muscles during inspiration, estimated using the following equation: (Peak airway pressure - Positive end-expiratory pressure (PEEP) x ((100 - Gain) / Gain). A grid built from this equation was available at the bedside ([Carteaux](javascript:void(0))), allowing the rapid estimate of the Pmus,Peak. From the values of the gain, the PEEP, and the Paw,Peak, which all are available on the screen of the ventilator, the Pmus,Peak can be immediately estimated.

The parameters at PAV initiation were set to a Gain of 50% and an inspiratory trigger of 2 l/min. The adjustment algorithm is defined as a function of the Pmus, peak. If Pmus, peak is less than 5 cmH2O, the gain is reduced in 10% steps until the pressurereaches the target range. If the pressure is higher than 10 cmH2O the gain is increased by 10% steps. If Pmus,peak is between 5 and 10 cmH2O no change is made unless the Vte exceeds 10 ml/kg of PBW without any obvious cause of hyperventilation justifying a decrease in gain by 10% steps or if the Vte falls below 5 ml/kg despite an optimization of FiO2 and PEP.

***Additional method 3. Quantification of patient-ventilator asynchrony***

The following three main patterns of patient-ventilator asynchrony were quantified: 1) ineffective triggering, 2) auto triggering, 3) double triggering. Premature cycling and late cycling could not be quantified because the analysis could not be done visually.

These patterns of asynchrony were quantified according to a detection methods based on flow and airway pressure signals only by visual inspection of the recordings.

The three main patterns of asynchrony were quantified off-line by the same investigators (C.B.), intensivists trained in detection of patient-ventilator asynchronies, who analyzed all the breaths. The investigator categorized each analyzed breath as "asynchrony" or "no asynchrony" according to the definition.

The asynchrony index (AI) was computed as the number of asynchronous breaths divided by the total number of breaths (both requested and delivered) multiplied by 100.

**Refrences**

1. Hug F, Raux M, Prella M, Morelot-Panzini C, Straus C, Similowski T. Optimized analysis of surface electromyograms of the scalenes during quiet breathing in humans. Respir Physiol Neurobiol. 2006;150(1):75-81.

2. De Troyer A, Kirkwood PA, Wilson TA. Respiratory action of the intercostal muscles. Physiol Rev. 2005;85(2):717-56.

3. Strohl KP, O'Cain CF, Slutsky AS. Alae nasi activation and nasal resistance in healthy subjects. J Appl Physiol Respir Environ Exerc Physiol. 1982;52(6):1432-7.

4. Schmidt M, Kindler F, Gottfried SB, Raux M, Hug F, Similowski T, et al. Dyspnea and surface inspiratory electromyograms in mechanically ventilated patients. Intensive Care Med. 2013;39(8):1368-76.

5. Fernández M, Pallás-Areny R. Ag-AgCl electrode noise in high-resolution ECG measurements. Biomed Instrum Technol. 2000;34(2):125-30.

6. Hewson DJ, Hogrel JY, Langeron Y, Duchêne J. Evolution in impedance at the electrode-skin interface of two types of surface EMG electrodes during long-term recordings. J Electromyogr Kinesiol. 2003;13(3):273-9.

7. Carteaux G, Mancebo J, Mercat A, Dellamonica J, Richard JC, Aguirre-Bermeo H, et al. Bedside adjustment of proportional assist ventilation to target a predefined range of respiratory effort. Crit Care Med. 2013;41(9):2125-32.

**Table S1. Differences of dyspnea, breathing pattern and blood gases between baseline (PSV-Baseline), after optimization of ventilator settings (PSV-Personalization) and with proportional assist ventilation (PAV)**

|  | Change between PSV-Personalization and  PSV-baseline | Change between PAV  and  PSV-Personalization | Change between PAV  and  PSV-baseline | *p* |
| --- | --- | --- | --- | --- |
| *Dyspnea* | | | | |
| D-VAS*, mm* | -13 (-26‒-8)* | -2 (-11‒0) | -17 (-30‒-10)* | 0.001 |
| IC-RDOS | -0.07 (-2.00‒0.01) | -0.02 (-015‒0.5) | -1.85 (-2.09‒0.01)* | 0.002 |
| *Breathing pattern* | | | | |
| RR, *min^‒1^* | -2 (-5‒0)* | -3 (-5‒0)* | -4 (-9‒-2)* | <0.0001 |
| Vt*, ml/kg IBW* | 0.8 (0.2‒1.9)* | 0.6 (-0.5‒2.2) | 1.7 (0.5‒2.6)* | <0.0001 |
| Ti*, sec* | 0.08 (-0.01‒0.18) | 0.17 (0.05‒0.25)* | 0.22 (0.13‒0.34)* | <0.0001 |
| Vt/ Ti*, L/s^‒1^* | 0.51 (0.10‒1.35)* | -0.95 (-2.29‒-0.29)* | -0.19 (-0.49‒-0.04) | <0.0001 |
| Pmax*, cmH_2_O* | 7 (6‒9)* | 4 (0‒7) | 11 (7‒14)* | <0.0001 |
| EtCO_2_*_,_ mmHg* | -1 (-2‒0) | 0 (-1‒2) | 0 (-1‒0) | 0.033 |
| *Breath-by-breath variability* | | | | |
| CV RR, *%* | 1 (-3–7) | 4 (-2–11)* | 6 (1–16)* | 0.001 |
| CV Vt*, %* | 0 (-5–3) | 10 (3–18)* | 10 (-1–25)* | < 0.001 |
| CV Ti*, %* | 3 (-3–15) | -13 (-23–1)* | -4 (-22–6) | 0.023 |
| CV Vt/ Ti*, %* | 1 (-7–11) | 0 (-14–27) | 2 (-9–32) | 0.632 |
| CV Pmax*, %* | 1 (-1–4) | 9 (5–17)* | 10 (6–17)* | < 0.001 |
| *Blood gases* | | | | |
| pH | 0.02 (0.01‒0.03)* | 0.01 (-0.01‒0.02) | 0.01 (0.00‒0.03)* | 0.012 |
| PaO_2_*_,_ mmHg* | 2 (3‒9) | 5 (-12‒14) | 6 (-1‒15)* | 0.016 |
| PaCO_2_*_,_ mmHg* | -2 (-2‒-1) * | 1 (-2‒3) | -1 (-3‒1) | 0.001 |
| SaO_2_*, %* | 0 (0‒1) | 1 (0‒3) | 2 (0‒2) | 0.038 |
| HCO^3-^*_,_ mmol/L* | 0.2 (-0.2‒0.3) | -0.1 (-0.3‒0.3) | 0 (-0.2‒0.2) | 0.503 |

D-VAS, Dyspnea visual analog scale, IC-RDOS, Intensive Care Respiratory Distress Observation Scale, RR, respiratory rate; Ti, inspiratory time; Vt tidal volume; IBW, ideal body weight; EtCO_2_, CO_2_ expired fraction; CV, coefficient of variation; Pmax, peak airway pressure

Data are expressed as median (interquartile range); * p <0.05

**Figure S1. Correlation between the dyspnea visual analog scale (D-VAS) and Intensive Care Respiratory Distress Observation Scale (IC-RDOS) in communicative patients (n = 18) at baseline (PSV-Baseline), after optimization of ventilator settings (PSV-Optimization) and with proportional assist ventilation (PAV)**

**Figure S2.** **Correlation between the coefficient of variation (CV) of descriptors of the breathing pattern and dyspnea assessed by the dyspnea visual analog scale (D-VAS, left panels) and Intensive Care Respiratory Distress Observation Scale (IC-RDOS, right panels)**


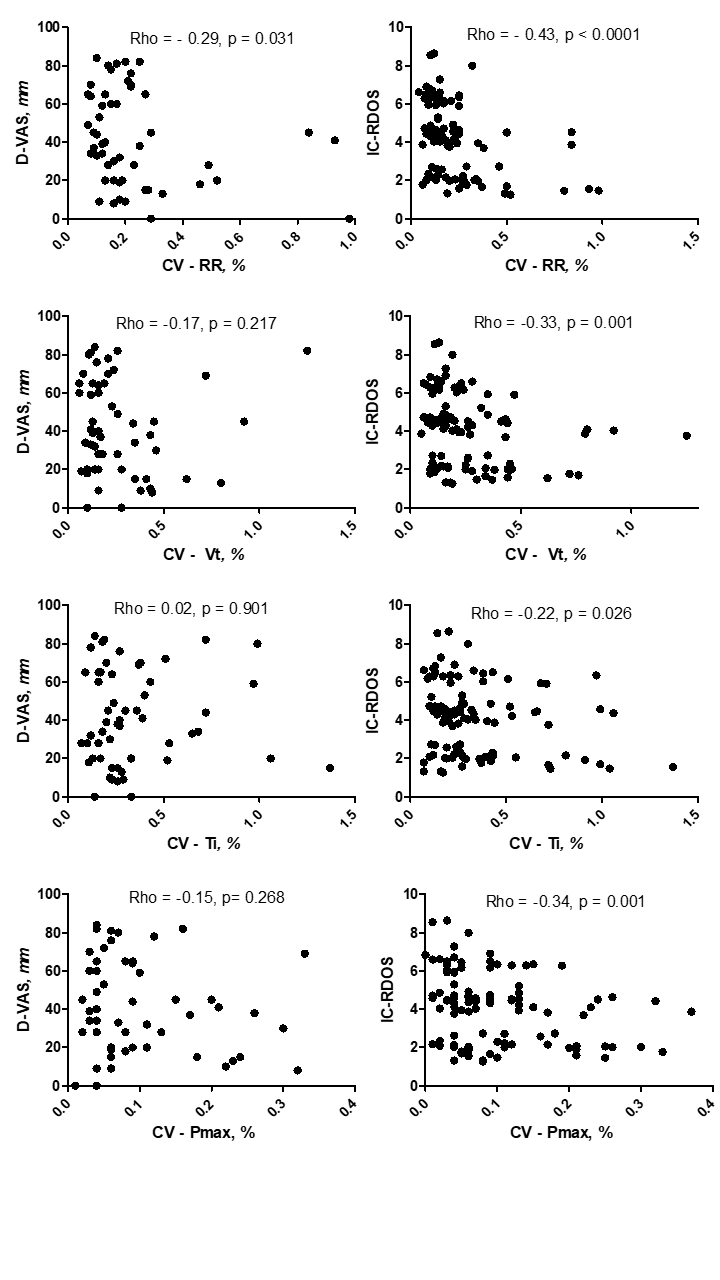


RR: respiratory rate, Pmax: maximal inspiratory pressure, Ti: inspiratory time, Vt: tidal volume

**Figure S3. Correlation between the pH and Intensive Care Respiratory Distress Observation Scale (IC-RDOS) in pressure support ventilation (A) and proportional assisted ventilation (B) and correlation between the bicarbonatemia and IC-RDOS in pressure support ventilation (C) and proportional assisted ventilation (D)**

**Figure S4. Prevalence of double triggering (Panel A), prevalence of auto-triggering (Panel B) and prevalence of ineffective triggering (Panel C) at baseline (PSV-Baseline), after personalization of ventilator settings (PSV-Personalization) and with proportional assist ventilation (PAV)**


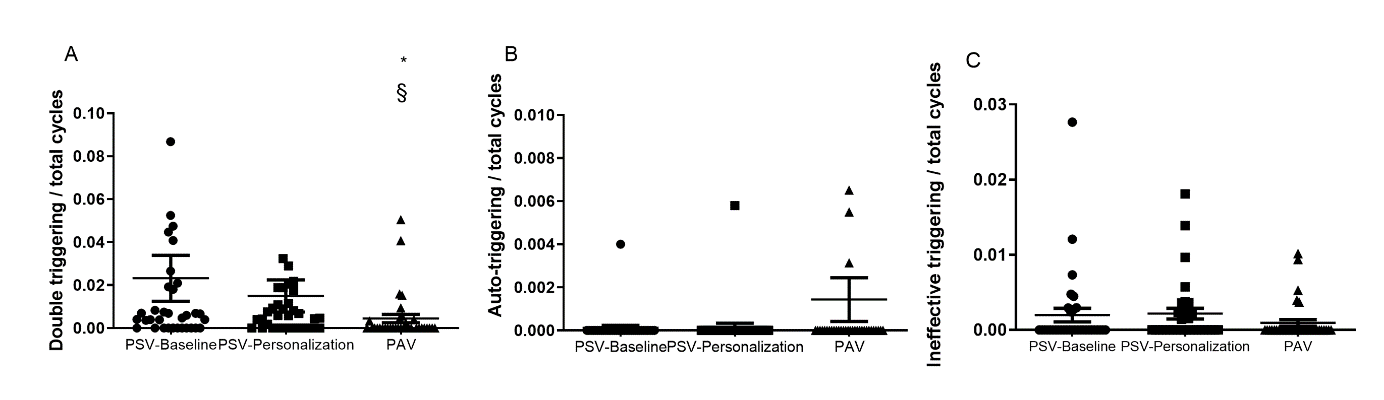


The horizontal solid line indicates median value.

* p <0.05 compared to PSV-Baseline, § p <0.05 compared to PSV- Personalization

**Figure S5. Correlation between the prevalence of double triggering and Intensive Care Respiratory Distress Observation Scale (IC-RDOS)**
